# Is decreased bone mineral density associated with development of scoliosis? A bipedal osteopenic rat model

**DOI:** 10.1186/1748-7161-6-24

**Published:** 2011-10-31

**Authors:** Ozgur Dede, Ibrahim Akel, Gokhan Demirkiran, Nadir Yalcin, Ralph Marcucio, Emre Acaroglu

**Affiliations:** 1 Hacettepe University Department of Orthopedics and Traumatology, Ankara, Turkey; 2 Izmir Kent Hospital, Cigli, Izmir, Turkey; 3 University of California San Francisco, San Francisco General Hospital, Department of Orthopedic Surgery, San Francisco, CA, USA; 4 Ankara Spine Center, Kavaklidere, Ankara, Turkey

**Keywords:** Idiopathic Scoliosis, Osteoporosis, Heparin, Rat model

## Abstract

**Background:**

An association between adolescent idiopathic scoliosis and osteopenia has been proposed to exist. It is still not clear whether there is such an association and if so, whether osteopenia is a causative factor or a consequence. Our previous pilot studies have suggested the presence of osteopenia in scoliotic animals. The aim of this study was to investigate the development of scoliosis in an unpinealectomized bipedal osteopenic rat model, implementing osteoporosis as a causative factor.

**Methods:**

Fifty Sprague-Dawley rats were rendered bipedal at the 3^rd ^postnatal week and separated into control (25 rats) and heparin (25 rats receiving 1 IU/gr body weight/day) groups. DEXA scans after 4 weeks of heparin administration showed low bone mass in the heparin group. Anteroposterior and lateral x-rays of the surviving 42 animals (19 in heparin and 23 in control groups) were taken under anesthesia at the 40^th ^week to evaluate for spinal deformity. Additional histomorphometric analysis was done on spine specimens to confirm the low bone mass in heparin receiving animals. Results of the DEXA scans, histomorphometric analysis and radiological data were compared between the groups.

**Results:**

Bone mineral densities of rats in the heparin group were significantly lower than the control group as evidenced by both the DEXA scans and histomorphometric analyses. However, the incidence of scoliosis (82% in heparin and 65% in control; p > 0.05) as well as the curve magnitudes (12.1 ± 3.8 in heparin versus 10.1 ± 4.3 degrees in control; p > 0.05) were not significantly different. Osteopenic rats were significantly less kyphotic compared to control specimens (p = 0.001).

**Conclusions:**

This study has revealed two important findings. One is that bipedality (in the absence of pinealectomy) by itself may be a cause of scoliosis in this animal model. Further studies on animal models need to consider bipedality as an independent factor. Secondly, relative hypokyphosis in osteopenic animals may have important implications. The absence of sagittal plane analyses in previous studies makes comparison impossible, but nonetheless these findings suggest that osteopenia may be important in the development of 3D deformity in adolescent idiopathic scoliosis.

## Background

Adolescent idiopathic scoliosis remains to be a major area of research. Many factors, such as proprioceptive defects, genetics, asymmetric or abnormal growth, soft tissue or neuromuscular conditions, have been scrutinized as potential causes [1-5], but none have been shown as a consistent factor in all scoliotic adolescents.

Several studies suggest a relationship between osteoporosis and scoliosis in adult patients [6,7]. Recurrent microfractures may lead to asymmetry, which would theoretically be augmented by the axial loading and this dependent cycle may result in a spinal deformity. Although has not been shown yet, the same pattern may apply to the developing spine of adolescents. Microfractures may result in an asymmetry which may propel with the aid of bone remodeling according to Hueter-Volkmann law, where the compressive side would diminish in growth, while the convex tensile side would have an enhanced growth. Bone density has been increasingly looked into since the 1980s after two reports indicated that children with adolescent idiopathic scoliosis had lower bone density when compared to their peers [8,9]. Sadat Ali et al. compared bone densities of girls with idiopathic scoliosis with their normal siblings and found significant differences [10]. However, it is not clear whether this finding is a cause or a result of the deformity. Another report by Cheng et al. indicated that children with idiopathic scoliosis indeed had lower bone density, but the density did not correlate with the type or severity of the curves [11]. This finding may indicate that osteopenia is a causative factor rather than the result of the condition.

We previously conducted studies on chicken and bipedal mice models, where tamoxifen was shown to alter the progression of scoliosis [12,13]. In the latter study, the spines of mice which had less deformity than the control bipedal group showed evidence of increased bone density but those results were not reported due to insufficient data. It seems probable that low bone mass may be an inciting or contributing factor in the development of spinal deformity in the bipedal animal models.

Clinical data supporting the possible relationship of osteoporosis or osteopenia and adolescent idiopathic scoliosis is confusing [14-16]. Our literature search failed to show any animal studies directly looking into the effects of osteoporosis or low bone mass (to the best of our knowledge, there is no set criteria for osteoporosis in rats to assess fracture risk, for that reason we believe the terms 'low bone mass' or 'decreased bone mineral density' are more appropriate) on the development of spinal deformity in the immature skeleton. Therefore, the objective of the current study was to investigate the effects of decreased bone mineral density (BMD) on the alignment of developing rat spine.

## Methods

**Figure 1:**
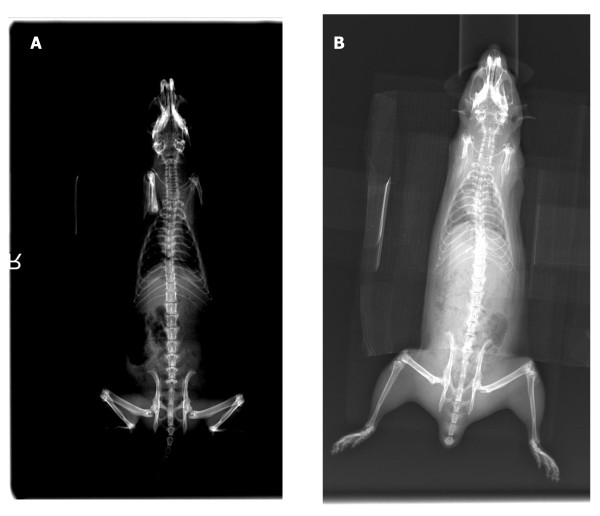
**Typical AP xray films of control (A) and heparinized (B) rats**. Heparinized rats showed slightly higher magnitudes of spinal deformity, although not statistically significant.

The experimental protocol was approved by Hacettepe University Medical Research Institution and conducted at the live animal research facility of the same institution. 50 female Sprague-Dawley rats were acquired from the Research Animals facility of Hacettepe University School of Medicine. The facility houses the rats in climate and photoperiodically controlled rooms. After weaning from their mothers at 3 weeks of age, all rats were rendered bipedal by amputating the forelimbs at a high humeral level and tails at the root under anesthesia as previously described [17,18]. Then, the rats were separated into 2 groups: 25 bipedal rats received no medication (control group) and 25 bipedal rats received daily subcutaneous heparin sodium injections (heparin group). A second, or 'true', control group consisting of normal (quadrupled) animals was not used since the literature supports that there is naturally no spinal deformity in quadrupled rats [17,18]. Heparin injections were started 5 days after surgery to give animals time to recover from the procedure. Healing during this period allowed for epithelization and prevention of hemorrhage from the amputation stumps. Daily heparin dose was 1 IU per gram of body weight of the rat. Dosage was determined according to previous publications showing daily heparin injections induce osteoporosis in rats [19-22]. Daily heparin injections were continued until the rats were euthanized. The animals were kept in standard cages (5 per cage) that allowed free in-cage motion and were fed ad libidum. Food and water were provided at the top of the cages and the cage size was increased gradually until the rats reached adult size in order to encourage more time standing erect. Dual energy X-ray absorbtiometry (DEXA) scans were done on a GE LUNAR 8743 BX-1L (Madison, WI, USA) device at the 4th week of heparin injections to evaluate the bone density. DEXA scans were done using the small animal protocol of the device's original software. Overall bone density and axial bone densities were compared using independent t-tests with significance set at p < 0.05. X-rays were taken on the 40th week to evaluate spinal alignment in both control and heparin groups A standard imaging position of the animals was established for taking x-rays by a custom made fixture, which prevents any rotation of the animals. The fixture was triangle triangular stand, each side angled 60 degrees from the horizontal, into which the rat's anterior chest wall was placed. This was done to be able to acquire a true anteroposterior x-ray. It is critical to avoid any out of plane rotation that could otherwise be caused by the rat's thorax morphology. This positioning is similar to the one described previously by Machida et al. [17]. Lateral x-rays were taken in a lateral decubitus position. Rats were put under anesthesia for the x-ray imaging procedure, which involved positioning them on the fixture as straight as possible without any applied traction. X-rays (Figure 1) were studied for the presence of any asymmetry. Additionally, spinal alignment was measured once by one of the authors (OD) using a DICOM viewing software digitally and then again manually on the printed copies using the Cobb method. Any measurable deformity was recorded and the results were compared between groups using independent t-tests. The minimum Cobb value we considered "measurable" was 3 degrees, since we do not believe that measuring a value less than this would be reliable. The number of scoliotic rats in both groups was also compared using Pearson's chi-square test. Any measurable angular deformity was included in statistical analysis.

Repeatibility of Cobb measurements were not analyzed for our data. A repeatability analysis comparing different observers was done at our institution by Turhan et al., on a chicken scoliosis model [23]. The authors used alpha scale reliability analysis to compare the interobserver repeatability of Cobb measurements and found that interclass correlations among observers were 0.8845 (95% confidence interval 0.8262-0.9270) for thoracic curves. An intraobserver analysis, however, was not done.

To evaluate the magnitude of osteopenia, 5 rat spines from both groups were dissected and preserved in EDTA (ethylenediaminetetraacetic acid) solution. The specimens were sent to San Francisco General Hospital Molecular and Cell Biology laboratory at the University of California, San Francisco for histomorphometric analysis. Lumbar vertebral segments of the rats were collected and fixed in 4% paraformaldehyde at 4°C for 24 hours. The samples were decalcified in 19% EDTA at 4°C for 10-14 days and then embedded in paraffin. Serial sections (10 μm) that were 600 μm apart along the entire vertebral body were stained with modified Milligan's Trichrome to visualize the cortical and trabecular bone. An Olympus CAST system was used to perform histomorphometry by applying Cavalieri's principle to determine the trabecular bone volume and total corpus volume of the same vertebral bodies of the rats. The volume of the bone was calculated using the equation for a conical frustum: 1/3 h (Ai+Ai+1+AiAi+1). *Ai *and *Ai*+1 are the area of bone in the sequential sections; *h *is distance between sections (600 μm); and, *n *is total number of sections analyzed for each specimen. Differences between groups were compared using a t-test.

## Results

Postoperatively and during follow-up, 6 rats from the heparin group and 2 rats from the control group died. Nineteen rats in the heparin group and 23 rats in the control group were available for evaluation at the end of the study. At 4 weeks DEXA scans showed significant differences between the bone densities of the control and heparin groups (p < 0.05) with the heparin group having lower bone density (Table 1).

**Table 1:** DEXA scan results of heparin treated and control rats show that daily subcutaneour heparin injections significantly decreased the local and overall bone mineral density

	Bone Mineral Density Mean ± STdev (gram/cm^2^)		
	**Heparin**	**Control**	**P value**

Legs	0.104 ± 0.014	0.114 ± 0.009	< 0.05

Pelvis	0.090 ± 0.011	0.100 ± 0.008	< 0.05

Spine	0.088 ± 0.011	0.102 ± 0.011	< 0.05

Total	0.110 ± 0.012	0.121 ± 0.008	< 0.05

Sixty-five percent of control animals, compared to 82% of osteopenic animals, developed scoliosis (p > 0.05) (Table 2, Figure 1). Average coronal curve magnitude was 12.1 ± 3.8 degrees in the heparin group whereas the control group was observed to be at 10.1 ± 4.3 degrees (Table 3) (p > 0.05). There were no statistically significant differences in the quantity and magnitude of the coronal curves between groups.

**Table 2:** Coronal curve incidence was not statistically significant between heparin and control groups.

		Group		
		**Heparin n(%)**	**Control n(%)**	**Total n**

Coronal curve rate	Absent	3 (%18)	8 (%35)	11
	Present	16 (%82)	15 (%65)	31

Total		19 (%100)	23 (%100)	42

**Table 3:** Magnitudes of coronal curves did not show a significant difference between groups, however mean sagittal curve magnitude of the heparin group was significantly lower than the control group.

	Group	n	Mean (degrees)	StDev	P value
Coronal	Heparin	16	12, 13	3, 757	0.167
	Control	15	10, 07	4, 284	

Sagittal	Heparin	19	59, 32	5, 726	0.001
	Control	23	65, 13	4, 137	

All rats had an acute kyphosis at the low thoracic level; however, rats in the heparin group were significantly less kyphotic compared to controls (59.3 ± 5.7 degrees and 65.1 ± 4.1 degrees, respectively; p = 0.001) (Table 3).

**Figure 2:**
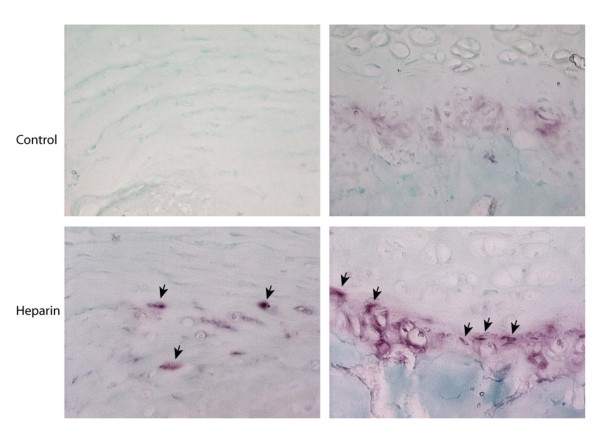
**Increased osteoclastic bone resorption (arrows) in the heparinized specimens**.

**Figure 3:**
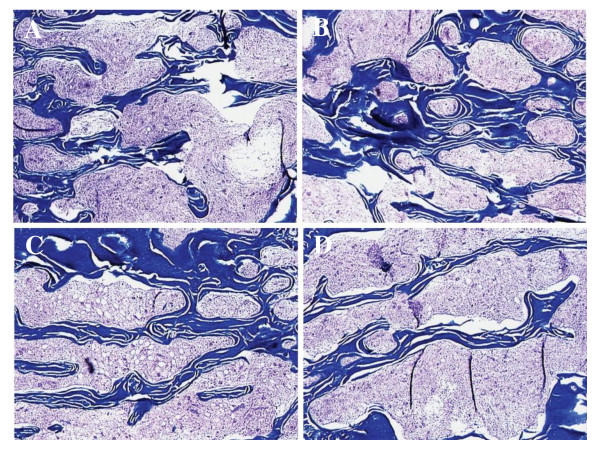
**A and B show control specimens where trabecular volume higher than the C and D, heparinized specimens showing the decreased trabecular morphology**.

Histomorphometric analysis revealed that both total vertebral body volume and trabecular volume were decreased in the heparinized group at the end of 40 weeks, which was statistically significant (p = 0.001 and p < 0.001, respectively) (Table 3). Figures 2 and 3 show the histologic and histomorphometric differences of spinal trabecular bone samples from heparin and control animals. In addition, the ratio of the trabecular bone to the total volume is found to be significantly decreased in the heparin group (p = 0.002) (Table 4).

**Table 4:** The comparison of total and trabecular bone volumes and the ratio of trabecular volume versus total volume for the lumbar vertebrae of the control and heparinized rats.

	Heparin	Control	P value (t-test)
Total Volume (mm3 ± StDev)	21.63 ± 2.22	27.74 ± 2.39	0.001

Trabecular Volume (mm3 ± StDev)	9.71 ± 0.67	15.96 ± 0.91	< 0.001

TrabV/TotV Ratio (% ± StDev)	45 ± 2.5	57.9 ± 6.2	0.002

Heparinized group demonstrated significantly low total and trabecular bone volumes. Note that trabecular to total volume ratio for heparinized rats is also significantly low compared to control animals.

## Discussion

This study investigated the effects of heparin-induced osteoporosis on the alignment of the developing spine in a bipedal rat model. Results demonstrated that the incidence of spinal malalignment at week 40 is not significantly different between the control group, which did not receive any medication, and the low bone mass group, which received daily subcutaneous heparin injections. DEXA scan results and histomorphometric analyses of spinal segments clearly show that daily heparin injections prove useful to decrease the trabecular bone density.

The death of experimental rats (six from heparin, two from control groups) decreased the number of specimens which may have decreased the statistical power of the findings. This may have contributed to the insignificance of the curve magnitudes between the groups.

Heparin, in addition to its anticoagulant effects, is of interest to orthopaedic surgeons because of its effects on bone healing and density. Since the 1960s, multiple clinical reports linked long term heparin use to osteoporosis [24-27]. There has been extensive research on topic and this relationship has been proven with clinical and laboratory studies on rats [19-22,28,29]. These studies showed an increase in osteoclast surface area and a decrease in osteoblast surface area thereby suggesting that Heparin causes osteoporosis by both increasing bone resorption and decreasing bone formation. Daily subcutaneous injections of Heparin causes 30% bone loss in rats at 28 days and continues even after heparin injections have been withdrawn. This effect of continued bone loss been linked to the sequestration of heparin molecules in bone tissue [30]. Daily heparin injections have minimal morbidity and proven efficacy in creating decreased bone mineral density. Therefore, in order to avoid the morbidity of additional surgery, we opted to administer daily heparin injections instead of ooferectomy. Our results are concordant with the available literature utilizing subcutaneous heparin injections.

Osteoporosis has previously been reported as a causative or a coexisting factor in idiopathic scoliosis patients in several studies [8,9,11,15,16,31,32]. These studies used DEXA scans and were able to show differences between scoliotic and age matched control groups. A recent study by Park et al. [33] looked into the osteogenic and adipogenic abilities of mesenchymal stem cells in 19 girls with AIS and 16 age- and sex-matched healthy control subjects. In children with AIS, the osteogenic differentiation ability of mesenchymal stem cell were lower when compared with controls whereas the adipogenic ability was not significantly different between groups. On the other hand, Szalay et al. [14] argued that the decreased Z scores in DEXA scans were related to the low body mass index of children with AIS and may not be related to scoliosis. They suggested that the decreased BMD in scoliosis patients is due to the lower BMI of these children and they mentioned that in their series scoliotic patients of normal and heavy weight demonstrated Z scores only slightly lower than the controls. However, their argument does not refute the theory that low BMD may be a contributing factor in development of scoliosis in children. However, currently available data strongly suggests a relationship between osteoporosis and AIS [34].

One major limitation of the current study is about the definition of 'osteoporosis' in rats. According to WHO criteria [35,36] osteoporosis is defined as a BMD value more than 2.5 standard deviations below the mean BMD of young adult women (BMD T-score < -2.5), and osteopenia (low bone mass) is defined as a BMD value between 1 and 2.5 standard deviations below the mean BMD of young adult women (-2.5 < BMD T-score < -1). The only study that defines osteoporosis in rats is by Sristava et al. [37] but the definition was only for categorical purposes and was not able to evaluate fracture risk. Therefore we chose not to use the term 'osteoporosis' but instead 'low bone mass', 'decreased bone mineral density' or 'osteopenia' to denote statistically significant differences. Heparin injections did cause a statistically significant decrease in BMD of the rats in all body regions. It is not known, however, whether this decrease is enough to cause clinically relevant problems, i.e. 'osteoporosis'. Another shortcoming of our study is that we did not use a placebo or sham injection group. We do not believe that subcutaneous injections of a placebo would be any different than no intervention at all, however, technically this is still a shortcoming of the study design. Also, it is of significance that the curve magnitudes are small and yet standard deviations are large which, makes the interpretation of the results difficult.

With the assumption that there is a relationship between osteoporosis and idiopathic scoliosis, one logical explanation could be that there is a treshold level of BMD needed for development of deformity and the BMD of our rats may have stayed below this threshold within the osteopenia region. Another argument could be that 40 weeks is not enough for the negative effects of osteoporosis to cause a significant change in vertebral bodies. Also, administration of heparin may have effects currently unknown to us. As an example, during the review process of this paper we have been informed by one of the reviewers that heparin has been shown to interfere with melatonin binding and signal transduction in chick brain [38]. This interaction has not yet been shown in rats, however the same effect maybe confounding our data. For this reason, we believe that another method to induce osteopenia, such as ooferectomy should be used to induce a more profound osteopenia which would also avoid the potential effects of a medication on the curve formation.

Our data implies that bipedality may be the major factor for the development of scoliosis in bipedal animal models and low bone mass may not significantly contribute to the degree of deformity in the developing spine. Yet, bipedality alone as a factor for the development of scoliosis is not supported by the literature. There are at least two other studies showing that rats that were rendered bipedal with a protocol very similar to the one used in this study did not develop scoliosis in the absence of pinealectomy [17,18]. It should be noted that these studies did not specify how they defined scoliosis. The disconcordance between these studies and the current study may be related to the differences in reporting threshold of the spinal deformity or simply due to differences in the protocols, such as the size of the cages, the amount of time animals spent on two hindlimbs, et cetera. The relationship of bipedality alone as an etiologic factor for spinal deformity should be further scrutinized with future studies.

Our study did not show a statistically significant relationship between low mineral density and development of scoliosis in a bipedal rat model. Another interesting finding of this study was that in heparin treated animals, a relative thoracic hypokyphosis was present. Considering that idiopathic scoliosis is a three dimensional deformity presenting with hypokyphosis in the sagittal plane, we believe that the relative hypokyphosis in the heparin treated animals maybe of some value. With these findings it is not possible to prove or refute the long questioned relationship between osteoporosis and idiopathic scoliosis. Further studies are required to clarify the relationship between osteoporosis and development of spinal deformity in the growing spine.

## Conclusions

Our findings suggest that osteopenia by itself may not be a major factor in the development of coronal deformity but may increase the incidence and severity, although not significantly. This study has revealed two other important findings. One is the fact that bipedality (in the absence of pinealectomy) by itself may be a cause of scoliosis in this animal model. Further studies on animal models will need to consider bipedality as an independent factor. Secondly, relative hypokyphosis in osteopenic animals may have important implications. Absence of sagittal plane analysis in previous studies makes comparison impossible but these findings suggest that osteopenia may be important in the development of the 3D deformity in idiopathic scoliosis.

## Abbreviations

IU: International Unit; EDTA: Ethylenediaminetetraacetic acid; DEXA: Dual energy X-ray absorptiometry; BMD: Bone mineral density.

## Competing interests

The authors declare that they have no competing interests.

## Authors' contributions

All authors have read and approved the final manuscript. Other contributions are OD: Laboratory work, surgery, care of the animals, drafting the manuscript, GD, NY: Laboratory work, surgery, care of the animals, IA: Laboratory work, surgery, statistics, drafting the manuscript, RM and EA: Study design, funding, final approval of the manuscript.
